# Muscle-Related Effect of Whey Protein and Vitamin D_3_ Supplementation Provided before or after Bedtime in Males Undergoing Resistance Training

**DOI:** 10.3390/nu14112289

**Published:** 2022-05-30

**Authors:** Yan Chen, Yiheng Liang, Hang Guo, Kun Meng, Junqiang Qiu, Dan Benardot

**Affiliations:** 1 Department of Exercise Biochemistry, Exercise Science School, Beijing Sport University, Beijing 100084, China; ryannchen98@gmail.com (Y.C.); liamyiheng5373@sina.com (Y.L.); guohang@bsu.edu.cn (H.G.); 2020110077@bsu.edu.cn (K.M.); 2 Department of Nutrition, Georgia State University, Atlanta, GA 30303, USA; dan.benardot@emory.edu; 3 Center for the Study of Human Health, Emory University, Atlanta, GA 30322, USA

**Keywords:** protein distribution, whey protein, vitamin D, muscle mass, muscle strength

## Abstract

There is increasing evidence that dietary protein intake with leucine and vitamin D is an important factor in muscle protein synthesis. This study investigated the combined effects of consuming whey protein and vitamin D_3_ in the evening before bedtime or in the morning after sleeping on muscle mass and strength. Healthy, untrained males (*N* = 42; Age = 18–24 year) were randomly assigned into three groups: before bedtime, after sleeping, and control. Subjects underwent a 6-week resistance training program in combination with supplements that provided 25 g whey protein and 4000 IU vitamin D_3_ for the before bedtime and after sleeping groups and a 5 g maltodextrin placebo for the control group. A significant increase in serum vitamin D was observed in both before bedtime and after sleeping groups. All groups experienced a significant gain in leg press. However, the control group did not experience significant improvements in muscle mass and associated blood hormones that were experienced by the before bedtime and after sleeping groups. No significant differences in assessed values were observed between the before bedtime and after sleeping groups. These findings suggest that the combination of whey protein and vitamin D supplements provided either before or after sleep resulted in beneficial increases in muscle mass in young males undergoing resistance training that exceeded the changes observed without these supplements.

## 1. Introduction

Muscle mass is regulated by a dynamic balance between muscle protein synthesis (MPS) and muscle protein breakdown (MPB) [1]. Resistance training (RT) in combination with dietary protein supplementation has been promoted as an effective approach to stimulate MPS, which is associated with muscle hypertrophy and elevated muscle strength [2,3,4]. Several factors impact the influence protein has on MPS, including the protein dose and protein quality, which is highly influenced by the relative amount of leucine in the protein consumed and the state of energy balance and hydration when the protein is consumed [5,6]. Dietary protein recommendations are typically presented in 24-h units, but it has become increasingly clear that the distribution of protein within each 24-h period is a critically important factor for sustaining and/or increasing musculature [7,8]. To maximize MPS an adequate protein quantity and quality per meal of 20–40 g (∼0.40 g/kg body mass) with 1–3 g leucine and 10–15 g essential amino acids (EAA) consumed when in a good energy-balanced state has been proposed [9,10,11,12]. Whey protein (hydrolysates and isolates) contains a high proportion of EAA and leucine with optimal solubility and digestion kinetics and has been extensively researched as a supplement in the RT population [8]. When compared with casein, which was preferred for pre-sleep ingestion, researchers reported that whey protein stimulated greater MPS at both rest and following exercise [13,14].

In addition to the quantity and quality of protein intake (PI), emerging issues include the timing and distribution of PI, which has seen increased research interest in recent years [15]. In general, the evenly distributed within-day pattern of PI is considered the optimal strategy for satisfying the total daily PI recommendation and for stimulating MPS [16]. However, regardless of the geographic region, it appears common for the distribution of PI to be skewed more heavily toward lunch and dinner [17,18]. Some studies have assessed the effect of a more evenly distributed PI by increasing the PI at breakfast, but long-term studies examining the impact of balanced distribution of PI have reported conflicting results [19,20,21,22]. For the RT population, studies have also reported that PI before sleep in the evening more effectively enhanced the MPS effects on muscle mass-related outcomes than consuming greater PI in the morning [8,9].

Low vitamin D (VD) status has become a global health issue, and in mainland China, the general population prevalence of VD deficiency is 20.7%, and insufficiency is 63.2% [23]. A recent meta-analysis has concluded that VD supplementation may beneficially affect muscle strength in young healthy women and men [24]. The impact of the combination of VD supplementation and RT on muscle quality (muscle strength/ muscle cross-sectional area) has been reported to a greater extent than the independent effects of RT in the elderly. Although results were non-significant, they indicated an increased functionality of the muscle to produce force [25]. Studies investigating the additive effect of VD on protein utilization found enhanced protein synthesis in murine C2C12 skeletal myotubes [26], but the effect of VD on MPS in humans is less clear [27].

Based on prior studies assessing positive muscle changes with modified PI and VD, it was the aim of this study was to examine the effectiveness of whey protein and VD_3_ supplementation on muscle outcomes in young males who are performing RT. A secondary aim was to assess the differential outcomes on muscle when protein and vitamin D supplements are consumed either before or after bedtime.

## 2. Materials and Methods

### 2.1. Subjects

Healthy untrained males (*N* = 58; 18–24 years) were made aware of this study through an internet advertisement and volunteered to participate. These individuals completed pre-screening tests, and 45 met the following inclusion criteria and were selected to participate: (1) healthy male college students with a normal body mass index (BMI); (2) no regular and organized exercise training over the past 6 months; (3) non-smoking status, no alcohol abuse, and no drug abuse; (4) had not taken any nutritional supplements in the past 6 months or longer; (5) lack of long-term outdoor activities or not exposing a significant proportion of skin to sunlight during outdoor activities; (6) consuming adequate daily dietary protein (>0.80 g·kg^−1^·d^−1^); and (7) had a serum VD level suggestive of deficiency (serum 25(OH)D concentration < 25 ng/mL). 

The study protocol was conducted in compliance with the declaration of Helsinki and approved by the Internal Review Board of Beijing Sport University (NO. 2021151H). Subjects were fully informed of the experimental procedures and potential risks and benefits of the study protocol, and voluntary signed informed consent was received from all participating subjects. Of the 45 subjects who initiated the study, 42 completed all study procedures. A total of three subjects were removed from the protocol because of scheduling conflicts, non-compliance in taking supplements, and accidental injury.

Subject characteristics are shown in Table 1. At baseline, there were no significant differences between the groups for age, height, weight, and serum 25(OH)D level. Serum 25(OH)D concentration of all subjects was lower than 25 ng/mL at pre-screening and when re-tested at baseline (week 2).

### 2.2. Study Design and Randomization Procedures

This 8-week study was a randomized, control, double-blind, parallel-group trial that included a 2-week pre-screening and baseline testing period (first phase) followed by a 6-week intervention period (second phase). The study was conducted from November to December 2021 in Beijing, China. During the pre-screening process, blood samples were collected for analysis of VD status, a self-reported dietary and lifestyle questionnaire was administered, and 3-day dietary records (including 2 weekdays) were obtained from all volunteer subjects. Those subjects who met the inclusion criteria were formally accepted to participate in the study. Randomization was stratified by serum 25(OH)D concentration and dietary PI to prevent any effect on outcomes of an uneven distribution over the group allocation. Random assignment of the subjects was conducted by an independent staff member not involved in the study, and study researchers and subjects were blinded as to the group allocation throughout the entire experimental period. A total of 45 subjects were assigned to the 3 groups: group 1, before bedtime (BB; *n* = 14); group 2, after sleeping (AS; *n* = 14); and group 3, control (C; *n* = 14).

**Figure 1:**
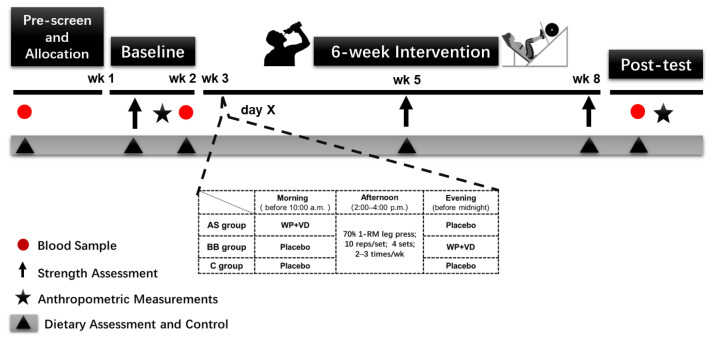
Experimental protocol.

All subjects were instructed to avoid consuming alcohol, additional caffeine supplements beyond normal food intake, and performing high-intensity exercise within 48 h prior to the tests. During the first phase (week 0–2) all subjects first participated in a progressive resistance training (RT) program (familiarization session) for two sessions during week 1 to learn how to perform the strength exercise correctly and to ensure an accurate strength assessment. The strength assessments were conducted at baseline (week 2), mid-intervention (week 5), and post intervention (week 8). During the 6-week intervention, subjects were required to consume whey protein (WP) and vitamin D (VD) supplements or a placebo, which consisted of maltodextrin (5 g) only at the assigned time every day. All subjects participated in the RT program 2–3 times per week for a total of 6 weeks, and the workload was set at 70% one-repetition maximum (1 RM), which was measured by the strength assessment and adjusted at mid-intervention (week 5). All subjects were asked to maintain their usual living and eating habits during the study, and dietary records were obtained throughout the entire trial period. Blood sample collections were conducted at pre-screen (week 0), baseline (week 2), and post intervention (week 8). Anthropometric measurements were performed to evaluate body compositions at baseline (week 2) and post intervention (week 8). (See Figure 1)

### 2.3. Supplement Intervention

Subjects of BB group consumed WP + VD nutritional supplements before bedtime and consumed the placebo in the morning, the AS group consumed the placebo before bedtime and consumed WP + VD nutritional supplements in the morning, and the C group consumed the placebo both before bedtime and in the morning. Subjects in the WP + VD group were asked to consume the nutritional supplements/placebo ~60 min prior to going to bed but before midnight regardless of sleep time and in the morning prior to 10:00 a.m. 

The nutritional supplement was in form of powder and each serving consisted of 25 g whey protein (Whey protein isolate Lacprodan^®^DI-9224, Arla Foods Ingredients, Viby, Denmark), 25 g maltodextrin (Glucidex^®^19, Roquette Frères, Lestrem, France), 4000 IU VD_3_ (Dry Vitamin D_3_^®^100 SD/S, Arla Foods Ingredients, Viby, Denmark), and added food flavor. The total energy provision of the supplement was 200 kcal per serving (50.01 g). The placebo was also in form of powder containing only maltodextrin (5 g; 20 kcal) and had flavoring to make it taste the same as WP + VD nutritional supplement. All supplements were in identical sachets and sequentially numbered corresponding with the subjects’ ID. Instructions for consuming supplements were also printed on the package. The instructions were as follows: “First, put 250 mL warm water in the cup, then pour all the supplement in, shake well, and drink”! Subjects were not allowed to consume any other nutritional supplements during the experiment period. The WP + VD nutritional supplement and placebo shared the same appearance, and the distribution of supplements was completed by staff who were not associated with the study. Both the experimenter and subjects were blinded to what supplement grouping each subject received, and the subjects could not identify the ingredients they consumed by packaging, appearance, or taste.

### 2.4. Dietary Assessment and Control

All subjects were instructed to keep daily food/beverage intake diaries by using a nutrient analysis application on their mobile phone (Boohee, Information Technology Co., Ltd., Shanghai, China) for the entire duration of the study. The use of Boohee app for quantifying food intake was previously shown to be an effective dietary monitoring tool [28]. During the pre-screen period, a 3-day food record was obtained using this application, including 2 weekdays and 1 weekend day, and was used to determine baseline energy substrate and caloric intake (% energy intake from carbohydrate, fat, and protein in each meal and snacks). During the 6-week intervention, subjects sent their food logs, including pictures of food, to the principal investigator every day and were also required to report when they consumed the provided supplements. All subjects were required to maintain their usual living and eating habits during the study except for test days, when standardized meals were provided.

### 2.5. RT Program 

The RT program was performed on a 45-degree leg-press machine (Technogym^®^, Gambettola, Italy), which was the same one used for the 1 RM test. In the familiarization sessions, all subjects were instructed and practiced on how to perform the leg press. All subjects completed progressive loading leg press for 3–5 sets at 50% estimated 1 RM load and took a simulated 1 RM test in the second session. 

During the 6-week intervention period, subjects participated in the RT program 2–3 times per week and a minimum of 16 sessions under the supervision of a qualified athletic trainer. Each training session was separated by at least 48 h. The RT program warm-up phase contained one set of 10 repetitions of 5 dynamic stretches (knee to chest stretch, lunge walk and leg swing, outside lunge and twist, dynamic squat stretch, and the world’s greatest stretch). The training phase contained 4 sets of 10 repetitions of leg presses at 70% of individual 1 RM load with a 60 s rest between 2 sets, which was followed by a cool-down phase. Individual 1 RM load was acquired in the 1 RM test at baseline (week 2) and adjusted after the mid-test (week 5). Training sessions were assigned at 2:00–4:00 p.m. in an indoor gym to assure that the time interval between RT and supplement intakes (before sleep) was more than 4 h. RT program attendance was recorded to assess compliance with the intervention and continued absence for 2 sessions would be removed from intervention.

### 2.6. Self-Reported Dietary and Lifestyle Questionnaire

A self-reported questionnaire collected information on lifestyle (living condition, sleep quality and habits), physical activity (type, intensity, duration, frequency, and place) and VD-specific food frequency. The questionnaire was translated from the English version of the VD-specific food frequency (i.e., high-dose VD-containing foods, according to the USDA national nutrient database) and lifestyle questionnaire (FFLQ) [29]. All data were collected online at baseline and post-test.

### 2.7. Strength Assessment

All subjects participated in two familiarization sessions in week 1 where proper techniques to perform the 45-degree leg press and knee extension, and the key points of strength assessment protocols were introduced. The familiarization RT program and the strength assessment protocol were all set for at least 48 h. All alcohol, drugs, nutritional supplements (except the supplements associated with the protocol), and high-intensity physical activities were avoided 48 h before and during test days.

#### 2.7.1. 1 RM Test

Maximum strength was assessed by a 1 RM test on a 45-degree leg-press machine. Through the familiarization sessions, subjects learned how to perform the leg press, and the individual machine settings were recorded for reproduction throughout the study. Subjects also completed a simulated test to obtain an estimated load that was used in the 1 RM test. In the formal 1 RM test, all subjects completed a warm-up consisting of one set of 10 repetitions of 5 dynamic stretches (same as in the RT program) and 1 set of 8 repetitions of 50% of the estimated 1 RM. After a 2 min rest, the load was progressively increased within 4–5 testing sets to reach the 1 RM at under 35 total repetitions, and every two sets were separated for at least 3 min. Each repetition began with knees extended, and then, subjects flexed their knees to reach 90°, and at any point of failure, the professional investigators re-racked the press and allowed the subject to rest for at least 3 min for another trial. If 1 RM was not acquired in five testing sets, the highest of the 3 RM values was used to estimate 1 RM [30]. Additionally, 3 RM value was assessed 48 h after the 1 RM test with the same procedure where the maximum of three consecutive repetitions was reached within five tests. Subjects whose 1 RM value was estimated by 3 RM took the same 3 RM test after the intervention. All tests were conducted by the same two experienced investigators, and verbal encouragement was provided. Importantly, no subject reported having experienced joint pain or muscle soreness due to the test.

#### 2.7.2. Isometric Knee Extension

Unilateral isometric knee extension (KE) was measured by a versatile device for knee extension (David F-200, David GmbH, Germany) equipped with a digital-analyzing module (MC-M; DAVID GmbH, Neu-Ulm, Germany) that records the real-time and peak torque during isometric muscle contraction. Subjects were required to adjust the seat height to position themselves properly, which facilitated muscle exertion. Subjects were fixed by the strap across the waist to ensure a static trunk thigh angle of 120° and a knee joint angle of 120°. The rotation axis of the lever arm was aligned to the lateral femoral epicondyle of subjects’ dominant leg, and the relative position of lever arms was selected by subjects. Both seat height and the position of lever arm were recorded to make assured all the conditions were the same in each subsequent test. Subjects performed 3-s maximal voluntary isometric KE three times with a 2-min interval [31]. The maximal torque observed during the tests was used in the statistical analysis.

### 2.8. Body Composition and Anthropometric Measurements

Anthropometric measurements to obtain body weight, body composition, and the sonographic anatomy of rectus femoris were performed at baseline (week 2) and post intervention (week 8). 

#### 2.8.1. DXA

Body composition and BMD were assessed with dual-energy X-ray absorptiometry (GE Lunar IDXA, Madison, WI, USA) using the standard procedure for a full-body scan. All subjects arrived at the laboratory in a fasting state on all test days in the morning and were asked to lie in the supine position without wearing any metal objects or electronic equipment to get a total-body scan. The instrument was calibrated with the standard calibration blocks by the same technician and preheated at the beginning of each test day. Using the enCORE version 15 software (GE Lunar, WI, USA), lean tissue mass (LTM), fat mass, bone mass (torso, upper limbs, lower limbs, and total body), and BMD index were automatically generated.

#### 2.8.2. B-Mode US

**Figure 2:**
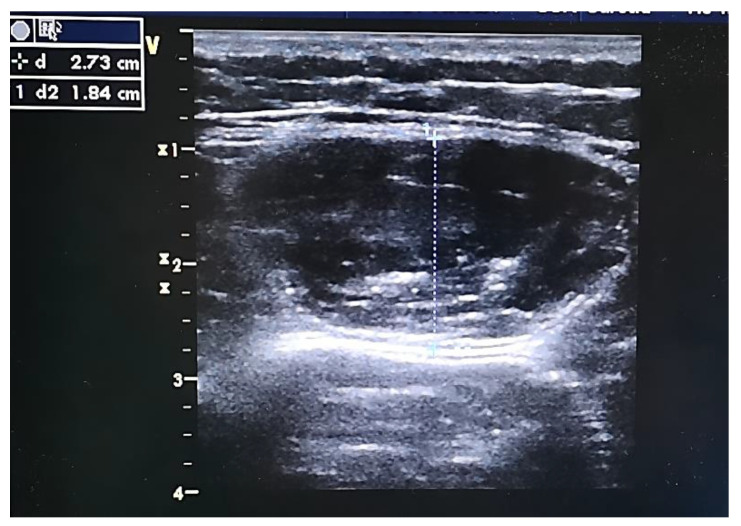
Ultrasonography picture of the RF.

B-mode ultrasonography system (GE Vivid 7; GE Medical Systems, Milwaukee, WI, USA) with a 13 MHz linear array transducer (GE 12L-RS; Milwaukee, WI, USA) was used to measure and calculate the thickness, circumference, and cross-sectional area (CSA) of the rectus femoris (RF). Subjects were positioned supine in bed with legs relaxed, hips and knees extended, arms resting comfortably at their sides, toes pointing the ceiling, and with a pillow behind their head to make sure the head was flat (0°). Using the anterior superior iliac spine and the upper pole of the patella as two reference points to form a line, the point of ultrasound measurement was marked with a pen at one-fourth of the distance to the patella on the dominant leg [32]. The probe was placed perpendicular to the skin on the thigh with minimal compression. The angle of the probe could be adjusted until the RF was clearly identified. Three to five images were taken and saved for analysis with the RF relaxed (see Figure 2). All ultrasonography measurements were performed by a single experienced investigator to eliminate between-measurer errors.

### 2.9. Blood Analysis

Blood samples were obtained three times from the antecubital vein at pre-screen (week 0), baseline (week 2) and post intervention (week 8). Venous blood was collected into 5 mL gel serum tubes in the morning (before 9:00 a.m.) after approximately 12 h overnight fasting and left to clot for 30 min at room temperature, after which the tubes were centrifuged for 10 min at 3000 rpm at 4 °C. Serum samples were stored at −20 °C until analysis. Serum 25(OH)D concentration was measured in all three blood samples with other blood indicators that included testosterone (T), cortisol, irisin, insulin-like growth factor-1 (IGF-1), and myostatin (MSTN), which were all measured at baseline (week 2) and after intervention (week 8). Serum 25(OH)D, irisin, IGF-1, and MSTN levels were measured using enzyme-linked immunosorbent assay (ELISA) kits (Jianglaibio, Shanghai, China) for quantitative detection with intra-assay coefficient variations (CV) < 9% and inter-assay CV < 11%. Serum T and C were measured with a fully automatic immunoanalyzer (Beckman DXC 800, Beckman Coulter, Fullerton, CA, USA).

### 2.10. Statistical Analysis

Power analysis was based on a previous study [33] on the effect of supplementing VD and whey protein at breakfast on muscle mass in the elderly, with a power of 80% and an α level (2-tailed) of 5%. We considered an expected mean increase of 0.5 kg in leg lean tissue mass in the AS and BB group and 0.2 kg in the C group and found that 12 subjects were required for each group to detect within- and between-group differences in the main outcome of leg lean tissue mass using DXA. Assuming an anticipated dropout rate of 20% during the intervention, 15 subjects were selected for each of the three groups. SPSS 25.0 software was used for performing statistical analysis. The distribution pattern of the data was tested using the Kolmogorov–Smirnov test, and all data were presented as means ± SD. A two-factor, mixed-design analysis of variance (ANOVA) with a between-subject factor (group) and within-subject factor (time) was used to evaluate the differences between and within the groups for outcomes. *p*-values of < 0.05 using 2-tailed tests were considered statistically significant. In case of a significant interaction, the simple main effects were tested and calculated to determine the difference between groups at each time point for each group. Otherwise, the main effects within the tests of within-subject effects were reported, and post hoc comparisons were performed via Tukey’s test. All graphs were plotted with GraphPad Prism 9 (GraphPad Software, San Diego, CA, USA).

## 3. Results

### 3.1. Nutrient Intakes

At baseline (Table 2), there were no significant differences between the groups for energy intake, total daily PI, and PI at each meal (*p* > 0.05). Daily PI of all subjects was over 0.80 g·kg^−1^·d^−1^, and as for each meal, all three groups consumed at least 0.24 g·kg^−1^ protein at lunch and dinner but not at breakfast (breakfast PI: AS group: 0.21 ± 0.09 g·kg^−1^; BB group: 0.21 ± 0.06 g·kg^−1^; C group: 0.20 ± 0.09 g·kg^−1^; *p* > 0.05). Within-day PI distribution skewed towards lunch and dinner at baseline in all subjects (breakfast vs. lunch vs. dinner: 0.21 ± 0.09 vs. 0.52 ± 0.23 vs. 0.61 ± 0.31 g·kg^−1^).

All subjects were required to maintain their former dietary habits during the entire study and, except for the WP + VD nutritional supplements that were provided as part of the study protocol, no other supplements were allowed. During the 6-week intervention period, there was no significant difference in total energy and nutrient intakes (protein, fat and carbohydrate) except for VD among the three groups. AS and BB groups consumed 101.8 ± 0.80 and 102.0 ± 1.09 ug VD daily compared with 2.1 ± 1.85 ug of C group (*p* < 0.001). Subjects of the AS group consumed 40.8 ± 6.66 g (0.49 ± 0.130 g·kg^−1^) protein at breakfast, and the distribution of PI over three meals tended to be balanced (breakfast vs. lunch vs. dinner: 0.49 ± 0.130 vs. 0.53 ± 1.460 vs. 0.53 ± 0.125 g·kg^−1^), whereas in the BB and C group, the PI distribution still skewed to lunch and dinner (see Table 3).

### 3.2. Body Composition and Anthropometry

**Figure 3:**
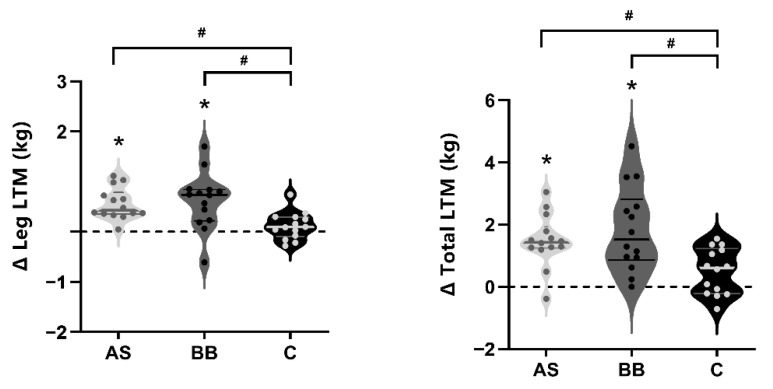
The violin plots of changes in leg muscle and total lean mass in AS, BB, and C group after 6-week intervention. The thick solid lines indicate the mean value of changes of each group, and the thin solid lines indicate interquartile range. An asterisk (*) indicates the change of the index is significant (*p* < 0.05). A pound sign indicates the difference between groups is significant (*p* < 0.05). A pound sign (#) indicates the difference between groups is significant (*p* < 0.05).

There were no significant differences in body composition-related measures between groups at baseline (Table 4). During the 6-week nutritional supplements combined with RT intervention, significant changes over time in body weight, fat mass, total lean tissue mass (LTM), leg LTM, and appendicular LTM (*p* < 0.05) were observed with no statistically significant differences between groups. Fat mass and fat mass% decreased significantly (*p* < 0.05) in AS group (change of fat mass: −0.718 kg) and BB group (−1.070 kg), and the decrease of fat mass% in the BB group was significantly greater than that of the C group (−1.5% vs. −0.514%; *p* < 0.05). Significant increases occurred in leg-LTM (AS: +0.556 kg; BB: 0.618 kg; *p* < 0.05) and total LTM (AS: +1.488 kg; BB: +1.847 kg; *p* < 0.05) in both the AS and BB groups. The changes in leg-LTM and total LTM of the AS and BB group were significantly higher than those of the C group (*p* < 0.05) (Figure 3). Total LTM% at post intervention (week 8) was significantly higher in the BB and AS groups than that of the C group (*p* < 0.05), and there was no significant difference between the mean of AS and BB groups.

**Figure 4:**
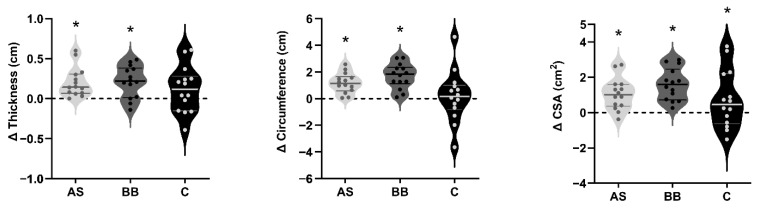
The violin plots of changes in the thickness, circumference, and cross-sectional area (CSA) of the rectus femoris (RF) in AS, BB, and C group after 6-week intervention. The thick solid lines indicate the mean value of changes of each group, and the thin solid lines indicate interquartile range. An asterisk (*) indicates the change of the index is significant (*p* < 0.05).

The thickness, circumference, and cross-sectional area (CSA) of the rectus femoris (RF) were measured through a B-mode ultrasonography system. There were significant changes in thickness, circumference, and CSA of rectus femoris (*p* < 0.05) for time, and no statistically significant difference was found between groups (*p* > 0.05). The change in the thickness, circumference, and cross-sectional area (CSA) of subjects in AS and BB groups were statistically significant (*p* < 0.05). The C group only experienced a significant change in CSA (*p* < 0.05) (see Figure 4). 

### 3.3. Blood Hormones 

**Figure 5:**
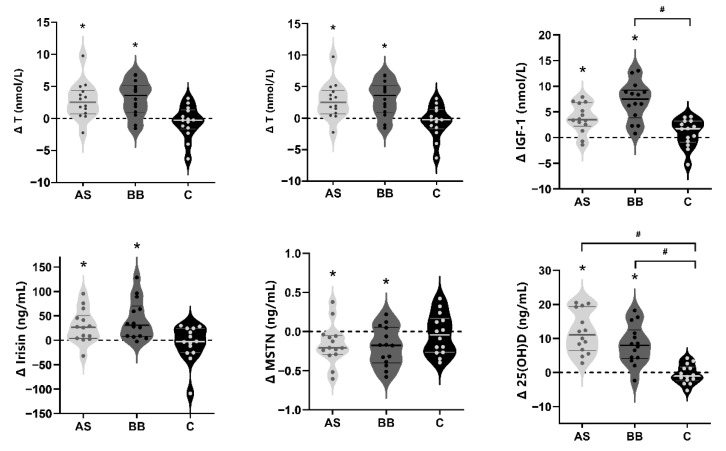
The violin plots of changes in the blood hormones in AS, BB, and C group after 6-week intervention. The thick solid lines indicate the mean value of changes of each group, and the thin solid lines indicate interquartile range. An asterisk (*) indicates the change of the index is significant (*p* < 0.05). A pound sign (#) indicates the difference between groups is significant (*p* < 0.05).

A significant increase from week 2 to week 8 in serum T levels occurred in AS and BB groups (*p* < 0.05) but not in the C group (*p* > 0.05) (Figure 5). There was a significant change in cortisol levels over time (*p* < 0.05). The cortisol levels at week 8 in the BB group were lower than those in the C group (*p* < 0.05), but there was no significant difference in mean values or changes in cortisol levels between AS and BB groups (Table 5). The changes in IGF-1 concentrations in AS and BB groups from week 2 to week 8 were significant (*p* < 0.05), and the IGF-1 levels of the BB group were significantly higher than that of the C group (*p* < 0.05) (Figure 5). As for the serum level of two muscle mass-related myokines, a significant increase was observed in irisin and MSTN in AS and BB groups (*p* < 0.05), but no significant changes were found in the C group (*p* > 0.05).

At weeks 0 and 2, serum 25(OH)D concentrations did not differ within and between the three groups (week 0: 14.9 ± 5.63 ng/mL; week 2: 13.9 ± 5.53 ng/mL; *p* > 0.05). After 6-week intervention, the concentrations of serum 25(OH)D of AS group (+11.871 ng/mL; 95% CI, 8.328 to 15.415 ng/mL) and BB group (+5.659 ng/mL; 95% CI, 2.116 to 9.203 ng/mL) increased significantly (*p* < 0.05). At week 8, 25(OH)D levels of the AS and BB group were significantly higher than that of the C group, but no significant difference was found between AS and BB group (*p* > 0.05).

### 3.4. Muscle Strength

**Figure 6:**
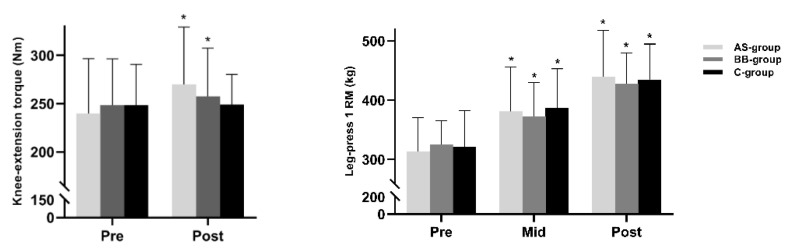
The bar plots of knee-extension (KE) peak torque and leg press 1 RM values in AS, BB, and C group pre, (mid), and post 6-week intervention. Data are presented as mean ± SD. An asterisk (*) indicates the change of relative index is significant (*p* < 0.05).

A significant change in leg-press 1 RM values (*p* < 0.05) and knee-extension peak torque (*p* < 0.05) occurred over time (Figure 6). However, there was no significant difference between groups observed in either of the assessments. In the leg press 1 RM test, consistent gradual increases occurred throughout the 6-week RT program in all three groups and the increase of 1 RM value was 125.8 kg (AS = 95% CI, 98.4 to 153.2), 103.2 kg (BB = 95% CI, 41.5 to 68.2), and 113.3 kg (C = 95% CI, 83.6 to 143.1). In knee-extension test, the increase in AS group (+29.929 Nm; 95% CI, 10.419 to 49.438) and BB group (+9.643 Nm; 95% CI, −9.643 to 29.153) were statistically significant (*p* < 0.05) but not in C group (+0.714 Nm; 95% CI, −18.795 to 20.224). There was no significant difference between AS and BB group in muscle strength assessments.

## 4. Discussion

To our knowledge, this randomized, controlled, parallel-group trial was the first study conducted on young males undergoing resistance training that evaluated the effects of simultaneously consuming whey protein and VD_3_ at different times on muscle mass and strength. Significantly greater muscle gain (leg-LTM, app-LTM, and LTM) and relative muscle mass gain (LTM%, app-LTM%) in AS and BB groups were observed, but no significant increase in these variables was observed in the C group. In addition, there were no significant differences in any parameter (i.e., body composition, muscle strength, and blood parameters) observed for subjects whose nutritional supplementation was consumed before bed time (in BB group) or in the morning (in AS group).

To date, several epidemiological studies [20,22,34] and intervention studies [35,36] have reported that dietary protein intake is one of the most important factors for the regulation of muscle mass. In the current study, volunteers whose total dietary protein intake reached at least 0.80 g·kg^−1^·d^−1^ before intervention were eligible to participate in the formal study. This inclusion criterion was based on the international society of sports nutrition position stand [8], where 0.80 g·kg^−1^·d^−1^ of “good-quality” protein denotes the recommended dietary allowance (RDA) for healthy adults [7]. According to the 3 days dietary record (2 weekdays and 1 weekend day), the subjects consumed 1.28 ± 0.49 g·kg^−1^·d^−1^ relative protein mass with no significant difference between groups at baseline. Based on the position stand, to maximize muscle protein synthesis in the population undergoing resistance training a protein intake of 1.6 g·kg^−1^·d^−1^ is recommended. During the intervention period in our study, subjects of AS and BB groups consumed over 1.50 g·kg^−1^·d^−1^ protein (including WP + VD supplementation), which approached the recommended level.

Because muscle protein synthesis has a saturable dose relationship with the quantity of dietary protein consumed, there is a shift in focus on protein intake from daily intake to individual meal intakes [7,16]. Several studies have confirmed that a more balanced protein distribution within meals and throughout the day is associated with more favorable MPS outcomes [19,20,21,22,37]. In our study, the protein intake skewed towards lunch and dinner at baseline in all subjects, and PI at breakfast in all three groups was lower than 0.25 g/kg/meal and protein intake at lunch and dinner were more than 0.5 g/kg/meal. A dose of PI of 0.25 g/kg/meal was reported as the minimum recommended PI per meal through the study by Moore et al. [38], and 0.4 g/kg/meal would optimally stimulate MPS [7]. In addition to insufficient breakfast protein intake, more than three-quarters of the subjects in our studies reported they would not consume breakfast unless they had morning lessons at 8:00 a.m., which meant they only consumed breakfast 2–3 days a week considering their schedules. The uneven distribution of protein intake in three meals, which was more favorably skewed toward lunch and dinner, has been reported by many studies [34,39,40,41]. Studies assessing Japanese [42] and USA [43] populations have reported similar findings, indicating that the dose and proportion of PI for breakfast were the lowest of the three meals and were much lower than the recommended PI for each meal (0.25 g/kg BW). Jun Yasuda et al. (2019) [34] concluded that breakfast with a high dose of protein may be the key to the regulation of muscle mass under free-living conditions, and in individuals whose daily PI did not reach 0.61 g/kg lean tissue mass, breakfast protein intake was significantly associated with RT-induced lean tissue mass gain.

Other studies focused on protein supplements also found that since subjects could consume enough protein at lunch and dinner, the importance of protein intake at breakfast should be emphasized to achieve the ideal total protein intake and balanced protein intake distribution [9,44]. In this study, subjects of the AS group who took WP + VD nutritional supplements (containing 25 g whey protein) after overnight sleep in the morning consumed 40.8 ± 6.66 g (0.49 ± 0.130 g/kg BW) protein at breakfast for 6 weeks according to their daily dietary record. The distribution of protein intake in AS group tended to be evenly distributed over three meals (breakfast/lunch/dinner: 32/34/34%), whereas in the BB and C group, the ratios were 18/42/40% and 18/41/42%). However, there was no statistically significant difference in muscle mass and strength-related outcomes between the AS group and BB or C group before and after intervention at the average level. An 8-week dietary and RT intervention trial conducted by Kim et al. (2018) also showed no significant difference in lean tissue mass, muscle mass, and other functional outcomes between two different PI distribution patterns in mixed meals (Even: 33/33/33%; uneven: 15/20/65%) [45]. The mixed meals in the Kim et al. (2018) study were provided with protein-enriched food (i.e., eggs, dairy, beef), and the standardized dietary protein intake was found to be 1.1 g·kg^−1^·d^−1^, which was lower than in our trial. In our study, high-quality whey protein was provided, and all subjects consumed adequate protein (1.47 ± 0.399 g·kg^−1^·d^−1^) during the intervention period. We observed a significantly greater gain in absolute and relative muscle mass (LTM, app-LTM, and leg muscle) and significant changes in muscle mass and MPS-related hormones in both the AS and BB groups but not in the C group, which suggested the WP + VD nutritional supplements had positive effects on RT-induced muscle-related outcomes regardless of whether the distribution of dietary PI was or was not balanced. In general, this protocol suggests that exercise alone without any dietary intervention positively impacts strength-related outcomes, but an appropriate dietary intervention coupled with strength training exercise provides additional benefits.

Eating before bed time has long been controversial [46,47], but for the RT population aimed at muscle gaining, they preferred ingesting protein before bedtime, which has been identified as advantageous to MPS and muscle recovery in both acute and long-term studies among both adults and older adults [48,49,50]. Antonio et al. (2017) [9] conducted an 8-week intervention in healthy males to compare the effects of consuming protein (casein, 54 g per serving) in the morning before noon versus consuming protein ~90 min prior to sleep. This study found that the lean tissue mass of subjects in morning group increased by 0.4 kg, and the evening group increased by 1.2 kg despite no significant difference between groups, suggesting that supplementing casein before sleep could better stimulate MPS, which was also supported by data from Burk et al. [51]. Similar to our study, subjects of the BB group, who were required to ingest supplements ~30 min before sleep but before midnight to assure at least 7 h of sleep, gained 1.847 kg of lean tissue mass, and the AS group increased by 1.488 kg. Although no statistical differences could be identified in any outcomes we measured between the AS and BB groups, it was still of particular note because larger effect sizes were consistently reported in changes of the BB group compared with AS group. Our data suggested that the addition of WP + VD nutritional supplements, which provided 200 kcal per serving before bedtime, did not show adverse effects on body composition in the RT population.

In addition to whey protein, the intervention nutritional supplement used in this study also contained 4000 IU VD_3_. VD status assessed by serum 25(OH)D level has been confirmed to be significantly associated with muscle mass, strength, and function and exercise performance in adults [52,53,54]. The mean serum 25(OH)D concentration was lower than 25 ng/mL in the study population at baseline, while the normal range is defined as 30–50 ng/mL. VD insufficiency is defined as serum levels of 20–30 ng/mL [55]. Data suggest that, to maintain and develop muscle health, a higher serum level of 25(OH)D (25–50 ng/mL) should be reached [55,56,57,58,59]. Dzik et al. (2018) [60] found that daily supplementation of 3200 IU VD_3_ for 5 weeks can raise serum 25 (OH) D level by an average of 53 nmol/L (21.2 ng/mL) and may increase it above 85 nmol/L (34 ng/mL). The daily dose of VD of 4000 IU is usually defined as the tolerable upper intake level for adults [61], but this may also depend on practical circumstances. To correct VD deficiency, for instance, the Endocrine Society accepted that a daily intake of 10,000 IU VD may be needed [62]. For athletes and other populations engaging in high-level activities of daily living (ADL), studies have argued that 2000–6000 IU of VD should be consumed per day and, in some cases, even up to 10,000 IU [63]. In this study, VD levels of subjects in AS and BB groups significantly increased by 11.87 and 5.67 ng/mL, respectively with a supplemental intake of 4000 IU VD_3_. Considering this study was conducted in north China during winter, subjects who lacked outdoor activities could obtain little VD from sunshine-associated ultraviolet radiation B (UVB). Because of winter clothing, even if subjects spent significant amounts of time outside, the skin exposure would be limited so as not to contribute to serum vitamin D.

RT-induced increase in muscle mass and strength was largely based on changes in MPS [64]. Besides the direct impact through leucine-enriched whey protein, VD can also stimulate MPS through direct impact by increasing muscular VD receptor (VDR) level [65] and indirectly modulating the levels of anabolic hormones in blood [66,67]. Anabolic hormones serum testosterone (T) [68] and insulin-like growth factor 1 (IGF-1) [69] levels are associated with muscle mass and can help improve muscle adaptations to RT in male adults. Both RT and VD supplementation, alone or in combination, can increase serum T and IGF-1 levels, especially in VD deficient subjects [25]. We observed serum testosterone and IGF-1 increased significantly in the AS and BB group, but in the C group where only RT program was conducted, no gain was reported. Two muscle-mass-related myokines, irisin and myostatin, changed significantly in AS and BB groups. Data suggested that irisin is a good predictor of skeletal muscle mass [70], while myostatin is a negative regulator of muscle mass [71], and muscle strength is associated with circulating irisin and myostatin levels [72]. Studies have confirmed that long-term RT intervention can help increase the serum irisin level and decrease the serum myostatin level, which was related to muscle gain [73,74]. In our study, the changes in blood hormone markers related to muscle health were consistent with changes in muscle mass and other body composition-related changes found in AS and BB groups. Furthermore, muscle strength assessed by knee extension significantly increased in these two groups. The magnitude of increases in muscle strength as well as the changes in body composition in our subjects were similar to the changes reported in other studies [38,67].

After 6 weeks of RT combined with nutritional intervention, subjects of AS and BB group showed significant gains in muscle mass and strength with beneficial changes of anabolic hormones in blood, while subjects who only performed the RT program without protein and vitamin D supplementation only obtained improvement in the leg press. However, contrary to our hypothesis, no difference in mean values or gains was found between AS and BB groups. Several studies aimed at comparing the effects of balanced and unbalanced protein intake distribution by increasing protein intake at breakfast to achieve a balanced distribution found no significant changes in muscle mass and muscle strength, and one of the explanations is that they did not consume enough protein and dose of protein matters more than distribution [45,75]. In our study, all subjects consumed sufficient protein, and there was no difference between groups. The BB group consumed supplements before sleep, which can be considered as adding a protein-enriched meal before sleep, resulting in a shorter fasting time between meals than the other two groups. An improved meal frequency can also contribute to an enhancement of MPS [8]. In general, consuming supplements either before bed time or in the morning resulted in positive effects on muscle health-related results.

Study limitations included the following: We did not have a group of VD or whey protein supplementation alone, making it impossible to identify whether protein alone or VD alone or protein combined with VD was responsible for the positive muscle effects that were observed. Only a few studies involving protein combined with VD ingestion have been carried out, mostly on older adults, and none of them analyzed the independent effects of each component. In addition, some reported no effects [67,76,77]. In the present study, we primarily wanted to compare the effects of different consumption timing and found positive results in both. In a future study, we will focus on whether the combined supplementation has superior results to the individual supplementation of either protein or vitamin D.

## 5. Conclusions

Consuming 6 weeks of whey protein and vitamin D_3_ supplements combined with resistance training in healthy young males showed beneficial effects on muscle mass and strength. Consuming protein and VD supplements before bedtime or in the morning resulted in similar benefits without any adverse effects on body composition. Our data suggest that undergoing an RT program with a non-nutrient placebo may achieve a moderate elevation in 1 RM muscle strength but without an elevation in muscle mass.

## Figures and Tables

**Table 1:** Subject characteristics at baseline.

	All Subjects (*N* = 42)	AS Group (*n* = 14)	BB Group (*n* = 14)	C Group (*n* = 14)	*F*	*P*
**Age**, y	21 ± 1.5	21 ± 1.7	22 ± 1.3	21 ± 1.4	0.737	0.485
**Height**, cm	177.3 ± 5.25	179.9 ± 5.21	175.3 ± 4.16	176.6 ± 5.47	3.27	0.490
**Weight**, kg	70.1 ± 9.32	72.2 ± 11.29	66.9 ± 7.31	71.3 ± 8.70	1.291	0.286
**BMI**, kg/m^2^	22.2 ± 2.24	22.2 ± 2.51	21.9 ± 1.94	22.5 ± 2.35	0.34	0.714
**25(OH)D**, ng/mL	14.3 ± 5.72	12.5 ± 3.91	16.1 ± 7.25	14.2 ± 5.34	1.424	0.253

AS, supplementation of whey protein and vitamin D in the morning after sleep; BB, supplementation of whey protein and vitamin D in the evening before bedtime; C, control group.

**Table 2:** Total dietary intake and dietary intake at each meal at baseline.

	All Subjects (*N* = 42)	AS Group (*n* = 14)	BB Group (*n* = 14)	C Group (*n* = 14)	*F*	*P*
**Energy intake**, kcal/d	1873 ± 503	1989 ± 396	1981 ± 341	2048 ± 622	4.15	0.24
**Protein**, g/d	92 ± 30	87 ± 20	82 ± 30	97 ± 33	2.874	0.068
**Protein**, g/kg BW/d	1.3 ± 0.49	1.3 ± 0.44	1.2 ± 0.46	1.4 ± 0.53	1.485	0.239
**Breakfast**	0.2 ± 0.09	0.2 ± 0.09	0.2 ± 0.06	0.2 ± 0.09	0.056	0.946
**Lunch**	0.5 ± 0.23	0.5 ± 0.22	0.5 ± 0.20	0.5 ± 0.27	0.962	0.351
**Dinner**	0.6 ± 0.31	0.6 ± 0.28	0.5 ± 0.20	0.6 ± 0.33	1.886	0.165
**Fat**, g/d	72 ± 24	79 ± 26	61 ± 17	75 ± 24	3.416	0.431
**CHO**, g/d	232 ± 78	253 ± 57	295 ± 56	249 ± 60	2.546	0.091

**Table 3:** Total dietary intake and dietary intake at each meal during intervention.

	All Subjects (*N* = 42)	AS Group (*n* = 14)	BB Group (*n* = 14)	C Group (*n* = 14)	*F*	*P* ^➂^
**Total ^➀^**						
**Energy**, kcal	2393 ± 467	2428 ± 487	2417 ± 449	2397 ± 461	3.21	0. 443
**Protein**, g	126 ± 28	130 ± 11	133 ± 21	117 ± 25	1.69	0.554
**Protein**, g/kg BW	1.6 ± 0.51	1.7 ± 0.27	1.7 ± 0.50	1.4 ± 0.37	1.221	0.361
**Fat**, g	85 ± 21	90 ± 19	80 ± 26	89 ± 25	2.989	0.334
**CHO**, g	270 ± 53	285 ± 56	281 ± 45	272 ± 54	3.315	0.182
**VD**, ug	68.6 ± 47.59	101.8 ± 0.80	102.0 ± 1.09	2.1 ± 1.85	263.991	<0.001
**Breakfast**						
**Energy**, kcal	541 ± 181	637 ± 120	450 ± 155	535 ± 109	20.529	<0.001
**Protein**, g	27 ± 15	41 ± 7	17 ± 8	22 ± 8	43.971	<0.001
**Protein**, g/kg BW	0.3 ± 0.12	0.5 ± 0.13	0.2 ± 0.14	0.2 ± 0.09	19.376	<0.001
**Fat**, g	18 ± 7	18 ± 6	17 ± 7	19 ± 7	2.436	0.101
**CHO**, g	68 ± 24	78 ± 18	58 ± 21	68 ± 15	17.209	<0.001
**Lunch**						
**Energy**, kcal	658 ± 151	711 ± 134	684 ± 154	706 ± 146	2.799	0.073
**Protein**, g	39 ± 10	38 ± 8	39 ± 9	38 ± 9	2.651	0.832
**Protein**, g/kg BW	0.5 ± 0.15	0.5 ± 1.46	0.6 ± 0.16	0.5 ± 0.12	1.862	0.731
**Fat**, g	26 ± 8	28 ± 8	22 ± 8	27 ± 7	2.436	0.101
**CHO**, g	79 ± 21	77 ± 19	83 ± 21	77 ± 17	4.126	0.213
**Dinner**						
**Energy**, kcal	746 ± 229	778 ± 153	697 ± 145	762 ± 210	3.294	0.048
**Protein**, g	39 ± 11	37 ± 7	40 ± 9	40 ± 12	3.045	0.059
**Protein**, g/kg BW	0.5 ± 0.18	0.5 ± 0.13	0.6 ± 0.16	0.6 ± 0.22	1.736	0.102
**Fat**, g	27 ± 10	30 ± 9	24 ± 9	28 ± 12	2.27	0.117
**CHO**, g	85 ± 27	90 ± 17	78 ± 19	89 ± 36	2.874	0.068
**Snacks ^➁^**						
**Energy**, kcal	397 ± 214	455 ± 214	372 ± 257	365 ± 152	0.474	0.628
**Protein**, g	17 ± 14	14 ± 9	20 ± 19	16 ± 9	0.388	0.683
**Fat**, g	16 ± 12	18 ± 10	17 ± 16	12 ± 7	0.525	0.598
**CHO**, g	46 ± 30	59 ± 35	36 ± 31	43 ± 19	1.494	0.244

^➀^ Total intake (protein, fat, CHO, VD) was calculated by the sum of intake for breakfast, lunch, dinner, snacks, and nutritional supplementation/placebo. ^➁^ “Snack” refers to food intake between meals. ^➂^ One-way ANOVA was used to evaluate the differences among groups.

**Table 4:** Changes in body composition.

		AS Group	BB Group	C Group	Group	Time	Group * Time
**Body weight**, kg	Pre	72.2 ± 11.29	66.9 ± 7.31	71.3 ± 8.70	0.244	0.043	0.8
	Post	72.9 ± 11.22	67.2 ± 9.04	71.9 ± 8.15			
	Change (95% CI)	0.756 (−0.193 to 1.705)	0.322 (−0.627 to 1.271)	0.626 (−0.323 to 1.575)			
**Fat mass**, kg	Pre	12.8 ± 5.35	12.0 ± 5.29	13.3 ± 5.50	0.676	<0.001	0.091
	Post	12.1 ± 5.12	10.9 ± 4.56	13.0 ± 5.23			
	Change	−0.718 * (−1.216 to 0.221)	−1.070 * (−1.567 to −0.573)	−0.287 (−0.784 to 0.210)			
**Fat mass** %	Pre	17.3 ± 5.05	17.5 ± 5.37	18.2 ± 5.37	0.782	<0.001	0.047
	Post	16.2 ± 4.95	16.0 ± 5.12	17.7 ± 5.14			
	Change	−1.11 * (−1.67 to −0.56)	−1.50 *# (−2.05 to −0.95)	−0.514 (−1.06 to 0.04)			
**Total LTM**, kg	Pre	56.2 ± 7.12	52.6 ± 4.21	55.1 ± 4.53	0.266	<0.001	0.003
	Post	57.7 ± 7.12	54.4 ± 4.54	55.6 ± 5.05			
	Change	1.488 * (0.940 to 2.036)	1.847 * (1.299 to 2.394)	0.5 (0.048 to 1.047)			
**Total LTM**%	Pre	78.4 ± 4.84	79.0 ± 5.49	77.7 ± 5.04	0.419	<0.001	0.001
	Post	79.6 ± 4.70	81.2 ± 5.22 $	77.6 ± 4.39			
	Change	1.21 *# (0.35 to 2.09)	2.2 *# (1.40 to 3.13)	−0.14 (−1.01 to 0.72)			
**Leg-LTM**, kg	Pre	19.1 ± 2.92	18.1 ± 1.54	19.7 ± 1.86	0.242	<0.001	0.004
	Post	19.7 ± 2.88	18.8 ± 1.54	19.9 ± 1.94			
	Change	0.556 *# (0.338 to 0.773)	0.618 *# (0.401 to 0.835)	0.117 (−0.1 to 0.334)			
**Leg fat mass**, kg	Pre	4.3 ± 1.68	3.8 ± 1.35	4.6 ± 1.70	0.439	<0.001	0.18
	Post	4.0 ± 1.63	3.7 ± 1.26	4.4 ± 1.63			
	Change	−0.276 * (−0.386 to 0.165)	−0.188 * (−0.298 to −0.077)	−0.13 * (−0.241 to −0.020)			
**App-LTM**, kg	Pre	25.9 ± 3.91	24.6 ± 2.10	26.3 ± 2.42	0.341	<0.001	0.078
	Post	26.6 ± 2.02	25.2 ± 2.07	26.6 ± 2.50			
	Change	0.759 * (0.463 to 1.054)	0.655 * (0.360 to 0.951)	0.3 * (0.005 to 0.595)			
**App-LTM** %	Pre	35.9 ± 2.41	36.9 ± 2.62	37.0 ± 2.24	0.446	0.009	0.208
	Post	36.6 ± 2.29	37.7 ± 2.41	37.1 ± 1.88			
	Change	0.64 * (0.03 to 1.25)	0.76 * (0.150 to 1.37)	0.04 (−0.57 to 0.65)			
**Bone mineral density**, g/cm^2^	Pre	1.27 ± 0.137	1.24 ± 0.102	1.22 ± 0.107	0.56	0.311	0.164
	Post	1.28 ± 0.130	1.23 ± 0.093	1.24 ± 0.110			
	Change	0.009 (−0.10 to 0.027)	−0.009 (−0.027 to 0.010)	0.016 (−0.002 to 0.035)			

A two-factor, mixed-design ANOVA was used to evaluate the differences between and within the groups. An asterisk (*) indicates the change of the index is significant (*p* < 0.05); a pound sign (#) indicates the difference of the change between groups (compare with C group) is significant (*p* < 0.05); a $ indicates the difference of the mean value between groups (compared with C group) is significant (*p* < 0.05).

**Table 5:** Changes in blood hormones.

	AS Group (*n* = 14)		BB Group (*n* = 14)		C Group (*n* = 14)		Group	Time	Group * Time
	Pre	Post	Pre	Post	Pre	Post			
**Testosterone**, nmol/L	19.3 ± 4.14	22.0 ± 5.80	18.6 ± 4.15	21.7 ± 4.78	20.2 ± 3.82	19.6 ± 4.79	0.901	<0.001	0.001
**Cortisol**, nmol/L	364.9 ± 47.45	315.1 ± 64.98	353.3 ± 73.16	295.4 ± 95.50 $	316.3 ± 85.18	372.9 ± 65.33	0.67	0.164	<0.001
**Insulin-likegrowth factor-1**, nmol/L	8.1 ± 5.98	11.9 ± 5.92	8.7 ± 4.17	15.8 ± 3.95 $	12.4 ± 5.39	13.3 ± 5.34	0.291	<0.001	<0.001
**Irisin**, ng/mL	154.5 ± 34.43	190.3 ± 54.92	156.8 ± 34.04	200.1 ± 69.87	171.7 ± 49.98	164.7 ± 44.30	0.828	0.001	0.009
**Myostatin**, ng/mL	0.54 ± 0.334	0.37 ± 0.294	0.58 ± 0.247	0.39 ± 9.293	0.51 ± 0.210	0.48 ± 0.280	0.895	0.002	0.237
**25(OH)D**, ng/mL	12.49 ± 3.91	24.37 ± 6.41 $	16.10 ± 7.25	21.76 ± 8.07 $	14.16 ± 5.34	13.67 ± 5.34	0.032	<0.001	<0.001

A two-factor, mixed-design ANOVA was used to evaluate the differences between and within the groups. An asterisk (*) indicates the change of the index is significant (*p* < 0.05); a $ indicates that the difference of the mean value between groups when compared with C group is significant (*p* < 0.05).

## Data Availability

Not applicable.
